# The chain-mediation pathway of gender regarding academic delay of gratification in college students is regulated by anxiety/depressive mood and prospective memory

**DOI:** 10.3389/fpsyg.2022.1015331

**Published:** 2022-12-08

**Authors:** Chen-Yang Jiao, Xun Song, Wen-Yi Shao, Lin-Pu Feng, Dong-Liang Jiao

**Affiliations:** ^1^School of Medical Imaging, Wannan Medical College, Wuhu, China; ^2^School of Mental Health, Bengbu Medical College, Bengbu, China

**Keywords:** anxiety, depression, prospective memory, academic delay of gratification, gender, mediation analysis

## Abstract

**Objective:**

This study investigated the relationship between gender and academic delay of gratification (ADOG) in college students and explored the mediating roles of anxiety/depressive mood and prospective memory to provide a theoretical intervention approach based on internal mechanisms.

**Methods:**

Random cluster sampling was conducted on 609 students from three universities situated in the Province of Anhui, China with the use of data from several questionnaires: the general information questionnaire, Generalized Anxiety Disorder Scale, Patient Health Questionnaire, Prospective and Retrospective Memory (PRM) Questionnaire, and ADOG Scale.

**Results:**

The females’ anxiety and depression levels were lower than that of the males, while the female PRM and ADOG performance improved when compared to that of the males. Anxiety and depression were negatively correlated with PRM and ADOG, respectively, whereas the PRM and ADOG data demonstrated a positive correlation. Depression/anxiety and prospective memory also played a chain intermediary role between gender and ADOG.

**Conclusion:**

Gender not only directly affects college students’ ADOG, but it also has indirect effects through depression/anxiety and prospective memory. Therefore, it is very important to treat students’ mental health differently according to gender to improve prospective memory and delayed academic satisfaction.

## Introduction

The academic delay of gratification (ADOG) represents the psychological tendency to delay immediate gratification or the impulse to pursue more valuable long-term learning goals. This concept was first introduced by Bembenutty and Karabenick (1998). The main idea of ADOG is that it enhances students’ ability for self-control and self-regulation. Moreover, it is translated into the maintenance of confidence and persistence while they face boredom and difficulties during the realization process of the overall academic goals. In that sense, they can actively resist possible temptations and concentrate on completing their studies. The previous survey Bembenutty et al. (2001) showed that ADOG was an important predictor of students’ evaluation for teachers’ teaching effect, which could significantly predict students’ academic performance. Students with higher ADOG demonstrated better self-efficacy, which was the reason for achieving greater academic performance (Bembenutty, 2002a).

It is due to the fact, that at this age the cognitive control system mediated by central nervous is relatively immature and so some of the brain functions are not fully developed. Consequently, ADOG can easily be affected by the internal and external environment (Anokhin et al., 2011). Therefore, it is of great significance to investigate the factors that influence students’ ADOG with an ulterior purpose to improve their academic performance (Zhuo, 2007).

Bembenutty and Karabenick (1998) reported that female college students had higher levels of ADOG than their male counterparts. The difference between males and females in the ADOG has yet to be sufficiently explained. There is no difference between males and females in intelligence level and learning ability. Therefore, we suspect that the differences in the cognitive styles of males and females may be influenced by external factors that lead to these differences in academic delayed gratification.

Academic delay of gratification can be affected by many factors. College students are found in the transition period between youth and adulthood, and their emotions are unstable. Family and school environment, the pressure exerted by the thought of future employment and other difficult situations impact their mental health, resulting in a high incidence of anxiety and depression (Regehr et al., 2013; Unwin et al., 2013). Studies have shown that negative emotions such as anxiety and depression harmed students’ studies (Awadalla et al., 2020). Moreover, alleviating anxiety and depression could help learners improve ADOG and devote themselves in a better way to the current learning tasks (Bembenutty, 2009). This suggested a negative correlation between negative emotions and ADOG.

Recent data indicated that negative emotions such as anxiety and depression led to adverse results regarding the prospective memory (Zhou et al., 2017). The prospective memory represents thinking focused on the future and more specifically involves planned activities and events in the future (Brandimonte et al., 2014). It is a cognitive activity, which is closely related to people’s daily life but also to self-control and metacognition. Impairment of the prospective memory will lead to the declination of self-control, which may affect ADOG (Beran et al., 2016). Therefore, it suggests that the prospective memory performing a cognitive function can be affected by individual and external factors, which may result in an adverse effect on ADOG along with emotional abnormalities.

At present, studies have found that males are more likely to be affected by anxiety and depression, which can result in learning problems (Derdikman-Eiron et al., 2012). For instance, a recent survey found that male college students in China suffer from more serious depression than female students (Gao et al., 2020). Based on the above research conclusions, the following hypothesis was put forward: depression/anxiety and prospective memory play a chain intermediary role between gender and ADOG, that is, gender → depression/anxiety → prospective memory → ADOG”.

In summary, this study constructed a chain-mediation effect model in accordance with the previous research results, and it examined the mechanisms between gender and ADOG in college students to explore the mediating roles of depression/anxiety and prospective memory in this process.

## Materials and methods

### Research objects

This survey was conducted in three universities situated in Anhui Province, China. The survey started on February 05, 2021 and finished on April 11, 2021. We collected 635 questionnaires through our survey, and 609 were valid, yielding a valid response rate of 95.91%.

### Sampling method

In the current online questionnaire survey, more than two classes were participated and randomly selected from each grade of every school. The distribution of the questionnaire links occurred *via* the QQ group or WeChat group. Each IP address is allowed to fill in only once. According to the Declaration of Helsinki, the study was conducted with the approval of the Ethics Committee of Bengbu Medical College. Before participating, each person checked a box indicating informed consent on an online questionnaire. Before each item was rated, participants were instructed on each scale and were invited to fill out self-report questionnaires anonymously and voluntarily, with the option to terminate participation at any time.

### Questionnaires

#### General information questionnaire

The questionnaire is a self-made questionnaire including general information (age, grade, gender, etc.).

#### Generalized anxiety scale

The scale was based on the quantitative evaluation criteria recommended by the Diagnostic and Statistical Manual of Mental Disorders, 5th Edition (DSM-5), published by the American Psychological Association (Toussaint et al., 2020). GAD-7 is an effective tool to identify possible causes of generalized anxiety disorder, as it represents a reliable and valid source in previous studies. The score was classified into 4 levels: 0–5, 6–9, 10–14, and 15–21 corresponding to non-mild, moderate and severe anxiety, respectively. In this study, the (Cronbach’s) α coefficient of the standardized item of the GAD-7 was 0.918, whereas the KMO test coefficient (Bartlett’s test, *p* < 0.05) was 0.902, indicating adequate reliability and validity of the scale.

The first item of GAD-7 is “Feeling nervous, anxious or on edge,” and the actual responses of the subjects were “not at all” in 238 (39.03 percent), “several days” in 270 (44.35 percent), “more than half the days” in 80 (13.06 percent), “nearly every day” in 21 (3.55 percent). The average score of this item was 0.81. The second item of GAD-7 is “Not being able to stop or control worrying,” and the actual responses of the subjects were “not at all” in 277 (45.64 percent), “several days” in 244 (40.00 percent), “more than half the days” in 65 (10.65 percent), “nearly every day” in 23 (3.71 percent). The average score of this item was 0.72.

#### Patient health questionnaire

PHQ-9 was based on the nine DSM-5 criteria, suggesting a high sensitivity to depressive symptoms (Levis et al., 2020). The obtained score was classified into five levels: 0–4, 5–9, 10–14, 15–19, and 20–27 corresponding to non-mild, moderate, moderately-severe, and severe anxiety, respectively. In this study, the Cronbach’s α of the standardized item was 0.905, whereas the KMO test coefficient (Bartlett’s test, *p* < 0.05) was 0.930. Those two coefficients showcased the high reliability and validity of the scale.

The first item of PHQ-9 is “Little interest or pleasure in doing things,” and the actual responses of the subjects were “not at all” in 239 (39.19 percent), “several days” in 293 (48.23 percent), “more than half the days” in 59 (9.68 percent), “nearly every day” in 18 (2.9 percent). The average score of this item is 0.78. The second item of PHQ-9 is “feeling down, depressed or hopeless,” and the actual responses of the subjects were “not at all” in 240 (39.35 percent), “several days” in 299 (49.19 percent), “more than half the days” in 51 (8.39 percent), “nearly every day” in 19 (3.06 percent). The average score of this item was 0.78.

#### Prospective and retrospective memory questionnaire

PRM Questionnaire was previously translated into Chinese and validated by researchers in China (Jing, 2013). The scale included prospective and retrospective memory. Specifically, retrospective memory was considered the basis for the realization of prospective memory. Therefore, the sum of those two memories reflected on the ability level of prospective memory. The scale included 16 items, with grade scores from 1 to 5 with the total score ranging between 16 and 80. When the score was higher, that was considered to lead to greater memory performance. In this study, the two coefficients showed reliability and validity of the scale, since the Cronbach’s α of the standardized item was 0.895 and the KMO test coefficient (Bartlett’s test, *p* < 0.05) was 0.889.

The first item of PRMQ is “Do they decide to do something in a few minutes time and then forget to do it?,” and the actual responses of the subjects were “very often” in 34 (5.65 percent). “quite often” in 98 (16.13 percent), “sometimes” in 352 (57.74 percent), “rarely” in 85 (13.87 percent), “never” in 40 (6.61 percent). The average score of this item was 3.00. The second item of PRMQ is “Do they fail to recognize a place they have visited before?,” and the actual responses of the subjects were “very often” in 37 (6.13 percent). “quite often” in 67 (10.97 percent), “sometimes” in 207 (33.87 percent), “rarely” in 203 (33.93 percent) “never” in 95 (15.65 percent). The average score of this item was 2.58.

#### Academic delay of gratification scale for college students

The ADOG scale was developed by Bembenutty et al. (2001), and was translated and revised into Chinese (Du, 2011). The ADOG scale included classroom ADOG and after-class ADOG. The questionnaire consisted of 10 questions, all of which were dilemma situations. The 4-level score was used to measure students’ ADOG ability by calculating the total score. In the case where the total score was higher, it was considered to correspond to the stronger ability of ADOG. The two coefficients (Cronbach’s α and KMO test) used in the current study were 0.853 and 0.865, respectively suggesting good reliability and validity of the ADOG scale.

The first item of ADOGS is “A. Go to a favorite concert, play, or sporting event and study less for this course even though it may mean getting a lower grade on an exam you will take tomorrow, OR B. Stay home and study to increase your chances of getting a higher grade,” and the actual responses of the subjects were “definitely choose A” in 57 (9.35 percent), “probably choose A” in 173 (28.93 percent), “probably choose B” in 169 (27.74 percent), “definitely choose B” in 210 (34.52 percent). The average score of this item was 2.87. The second item of ADOGS is “A. Study a little every day for an exam in this course and spend less time with your friends. OR B. Spend more time with your friends and cram just before the test,” and the actual responses of the subjects were “definitely choose A” in 36 (5.97 percent), “probably choose A” in 101 (16.61 percent), “probably choose B” in 136 (22.26 percent), “definitely choose B” in 336 (55.16 percent). The average score of this item was 3.27.

### Statistical analysis

The SPSS 23.0 software was used for the statistical analysis of this study. The measured data were expressed as Mean ± SD and the independent sample *t*-test is used to compare the means of two groups, that share common features. The Pearson correlation analyses were used to identify the relationships between variables, with a test level α of 0.05. The deviation correction method, with 5,000 bootstraps, was used to test the chain mediating effect.

## Results

### General demographic data of subjects

Among the respondents, 245 were males (40.23%) and 364 were females (59.77%). From the total number (609) of the participants, 92 of them were at the age of 18 or below (15.11%), 148 were aged 19 (24.30%), 150–20 (24.63%), 92–21 (15.11%); 57–22 (9.36%), while 70–23 and above (11.49%). 145 were in freshman year (23.75%), 131 were in sophomore year (21.46%), 145 were in junior year (23.89%), 127 were students in the fourth grade (20.88%), 61 students were in the fifth grade (10.03%).

### Estimation of the level of anxiety and depression of college students

The obtained data showed, that 18.07% of all students that enrolled on the study had anxiety above the moderate level (GAD-7 ≥ 10 points) while 24.15% had depression above the moderate level (PHQ-9 ≥ 10 points; Table 1).

**Table 1:** The level of anxiety and depression of college students.

GAD-7	Number (%)	PHQ-9	Number (%)
Normal (0–4)	301 (49.42)	Normal (0–4)	254 (41.71)
Mild anxiety (5–9)	198 (32.51)	Mild depression (5–9)	208 (34.14)
Moderate anxiety (10–14)	88 (14.46)	Moderate depression (10–14)	95 (15.61)
Severe anxiety (15–21)	22 (3.61)	Moderate and severe depression (15–19)	39 (6.41)
		Severe depression (20–27)	13 (2.13)

GAD-7, Generalized Anxiety Scale, PHQ-9, Patient Health Questionnaire.

### Studying the gender differences in anxiety, depression, prospective and retrospective memory, academic delay of gratification, grade, and age

There were significant gender differences in the anxiety, depression, PRM and ADOG among students. We observed higher GAD-7 and PHQ-9 scores in men than in women, whereas the values of PRM and ADOG in men were lower than those in women (Table 2). There was no difference in age and academic grade among them.

**Table 2:** Gender differences in GAD-7, PHQ-9, PRM, ADOG, grade, and age in the students.

	Male	Female	*t*	*p*
	(Mean ± SD)	(Mean ± SD)		
	*N* = 252	*N* = 357		
GAD-7	5.61 ± 0.31	4.71 ± 0.24	2.299	0.022*
PHQ-9	7.26 ± 0.38	5.95 ± 0.28	2.519	0.012*
PRM	51.42 ± 5.86	53.88 ± 8.77	−2.374	0.018*
ADOG	27.63 ± 6.71	29.69 ± 5.21	−3.906	0.000**
Grade	2.52 ± 0.62	2.59 ± 0.31	−0.673	0.501
Age	20.84 ± 0.54	20.88 ± 0.38	−1.31	0.191

GAD-7, Generalized Anxiety Scale; PHQ-9, Patient Health Questionnaire; PRM, Prospective and Retrospective Memory Questionnaire; ADOG, Academic Delay of Gratification Scale for College Students. *p < 0.05, **p < 0.01.

#### Correlation analysis of the GAD-7, PHQ-9, PRM, ADOG, grade and age respectively

The analysis showed that GAD-7 and PHQ-9 were negatively correlated to the PRM and ADOG, respectively, and PRM was positively correlated with ADOG. There was no correlation between other variables and Grade or Age (Table 3).

**Table 3:** Correlation analysis of the GAD-7, PHQ-9, PRM, ADOG, grade and age, respectively.

	Scores (Mean ± SD)	GAD-7	PHQ-9	PRM	ADOG	Grade	Age
GAD-7	5.087 ± 0.744	1					
PHQ-9	6.506 ± 1.565	0.740**	1				
PRM	52.893 ± 12.396	−0.167**	−0.215**	1			
ADOG	28.887 ± 6.270	−0.209**	−0.186**	0.414**	1		
Grade	2.58 ± 0.18	0.072	0.079	0.038	−0.043	1	
Age	20.85 ± 5.63	0.068	0.035	0.052	−0.008	0.805**	1

GAD-7, Generalized Anxiety Scale; PHQ-9, Patient Health Questionnaire; PRM, Prospective and Retrospective Memory Questionnaire; ADOG, Academic Delay of Gratification Scale for College Students. **p < 0.01.

### Mediation model and path analysis

#### The common-method bias test

Four scales (GAD-7, PHQ-9, PRM, and ADOG) were used to study the same group of subjects at the same time, therefore the common-method bias caused by the variation in it was easy to be applied. Therefore, the Harman single factor test in the common-method bias test was applied to all collected data in this study. The results showed that the initial eigenvalues of five factors were greater than 1 without rotation, while the variance explanation rate of the largest factor was 31.02% lower than the critical standard of 40%. Therefore, it can be determined that the data collected in this study conformed with the standard of the common-method bias test and no multi-collinearity was estimated.

#### Regression analysis

With ADOG as the dependent variable, for Analysis 1 the GAD-7, PRM, gender, age, and grade were selected as the independent variables, and for Analysis 2 the PHQ-9, PRM, gender, age, and grade were selected as independent variables to conduct the regression analysis. The results showed that GAD-7, PRM, and gender were the influencing factors of ADOG in Analysis 1, and PHQ-9, PRM, and gender were the influencing factors of ADOG in Analysis 2 (Table 4).

**Table 4:** Regression analysis (dependent variable, ADOG).

	Independent variables	*B*	SE	β	*t*	*P*	*X* ^2^	*F*	*P*
Analysis 1	GAD-7	−0.175	0.05	−0.13	−3.537	0	0.206	31.82	0
	PRM	0.195	0.019	0.382	10.412	0			
	Gender	1.417	0.48	0.107	2.951	0.003			
	Age	0.205	0.221	0.056	0.927	0.354			
	Grade	−0.489	0.308	−0.096	−1.587	0.113			
Analysis 2	PHQ-9	−0.097	0.043	−0.085	−2.28	0.023	0.196	30.015	0
	PRM	0.197	0.019	0.386	10.359	0			
	Gender	1.446	0.484	0.109	2.988	0.003			
	Age	0.187	0.222	0.051	0.84	0.401			
	Grade	−0.497	0.31	−0.098	−1.6	0.11			

GAD-7, Generalized Anxiety Scale; PHQ-9, Patient Health Questionnaire; PRM, Prospective and Retrospective Memory Questionnaire; ADOG, Academic Delay of Gratification Scale for College Students.

#### Chain mediation analysis

On the basis of the predictive effect of gender on college students’ ADOG, combined with two important variables, GAD-7/PHQ-9 and PRM, which are significantly related to ADOG, this study analyzed the path to ADOG and discusses the internal mechanisms behind the effects of gender on ADOG.

**Figure 1:**
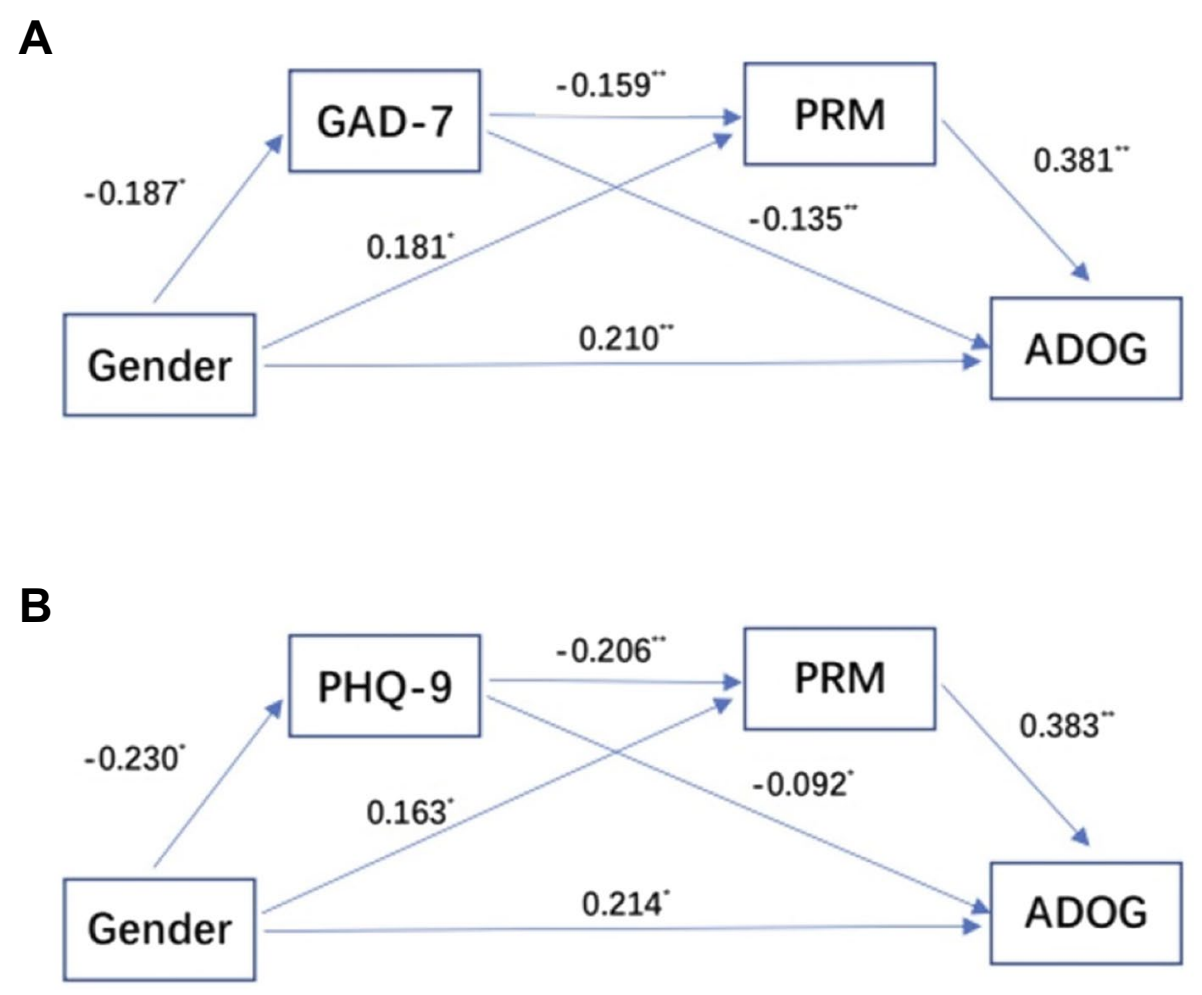
Chain-mediation pathway diagram. **(A)** Gender → GAD-7 → PRM → ADOG pathway diagram. **(B)** Gender → PHQ-9 → PRM → ADOG pathway diagram. GAD-7, Generalized Anxiety Scale; PHQ-9, Patient Health Questionnaire; PRM, Prospective and Retrospective Memory Questionnaire; ADOG, Academic Delay of Gratification Scale for College Students. *p < 0.05, **p < 0.01.

The deviation correction method, with 5,000 bootstraps, was used to obtain a 95% confidence interval to test the significance of the effects. If the confidence interval does not contain 0, then the statistical result is significant. As shown in Table 5 and Figure 1, gender had significant predictive effects on GAD-7/PHQ-9, PRM and ADOG, GAD-7/PHQ-9 had significant predictive effects on PRM and ADOG, PRM had significant predictive effects on ADOG.

**Table 5:** Chain mediation analysis.

Model	Intermediary variable	Dependent variable	Predictor variable	*R* ^2^	*F*	β	*t*
I	GAD-7 and PRM	GAD-7	Gender	0.009	5.283*	−0.187	−2.298*
		PRM	Gender	0.035	11.456**	0.181	2.239*
			GAD-7			−0.159	−4.006**
		ADOG	Gender	0.202	52.007**	0.21	2.846**
			GAD-7			−0.135	−3.707**
			PRM			0.381	10.389**
2	PHQ-9 and PRM	PHQ-9	Gender	0.013	7.989**	−0.23	−2.826*
		PRM	Gender	0.052	17.061**	0.163	2.037*
		ADOG	PHQ-9			−0.206	−5.209**
			Gender	0.192	48.894**	0.214	2.874*
			PHQ-9			−0.092	−2.474*
			PRM			0.383	10.310**

GAD-7, Generalized Anxiety Scale; PHQ-9, Patient Health Questionnaire; PRM, Prospective and Retrospective Memory Questionnaire; ADOG, Academic Delay of Gratification Scale for College Students. *p < 0.05, **p < 0.01.

The mediating effects, direct effects, and corresponding effect scales are shown in Table 6, and they indicated that a GAD-7/PHQ-9, PRM play intermediary roles between gender and ADOG.

**Table 6:** The 95% confidence interval of the mediating effect test and deviation corrections.

		Effect value	95% confidence interval		Effect ratio (%)
PATH			Bottom	Top	
1	1. Gender → GAD-7 → ADOG	0.165	0.018	0.371	8.05
	2. Gender → PRM → ADOG	0.447	0.034	0.905	21.79
	3.Gender → GAD-7 → PRM → ADOG	0.074	0.008	0.161	3.62
	Indirect effects	0.686	0.232	1.196	33.46
	Direct effects	1.365	0.423	2.307	66.54
	Total effects	2.051	1.021	3.083	
2	1. Gender → PHQ-9 → ADOG	0.138	0.006	0.322	6.73
	2. Gender → PRM → ADOG	0.407	0.015	0.866	19.84
	3.Gender → PHQ-9 → PRM → ADOG	0.118	0.029	0.233	5.75
	Indirect effects	0.663	0.206	1.156	32.32
	Direct effects	1.388	0.439	2.338	67.68
	Total effects	2.051	1.022	3.083	

GAD-7, Generalized Anxiety Scale; PHQ-9, Patient Health Questionnaire; PRM, Prospective and Retrospective Memory Questionnaire; ADOG, Academic Delay of Gratification Scale for College Students.

## Discussion

### College students have a high degree of anxiety and depression

Our results showed that 18.07% of college students had anxiety above the moderate level (GAD-7 ≥ 10) while 24.15% had depression above the moderate level (PHQ-9 ≥ 10). A recent study (Wang et al., 2021) investigating the prevalence of psychological disorders under the Covid-19 pandemics in China showed that anxiety above the moderate level (GAD-7 ≥ 10) accounted for 19.4% (a survey between January and March 2020), while depression was above the moderate level (GAD-7 ≥ 10) accounted for 24.8%. These data confirm our research results.

The emotional disorder screened by the scale was only the result of preliminary screening, and the specific situation needed to be further verified. However, the results of this survey showed that the college students had higher anxiety and depression disorders, which had an impact on learning.

### The significant gender differences in the generalized anxiety disorder scale, patient health questionnaire, prospective and retrospective memory, and academic delay of gratification among students

Our study found that there were gender differences in the GAD-7, PHQ-9, PRM, and ADOG among college students. The female GAD-7 and PHQ-9 scores were lower than those of the males, while the female PRM and ADOG performance improved when compared to that of the males. The survey was conducted during the coronavirus disease (COVID-19) outbreak, a stress factor that had different effects on the mood and learning of male and female college students in China. For example, studies have found that male college students had higher levels of depression during the COVID-19 lockdown (Gao et al., 2020). Also, females performed better than males in online learning during the COVID-19 lockdown (Liu et al., 2021). Therefore, the changes in learning methods that were triggered by the COVID-19 lockdown were less likely to affect females than males (Hsiao, 2021). Bembenutty (2002b) reported that gender was a significant predictor of the ADOG of college students, with female students reporting higher levels of ADOG than their male counterparts. These conclusions are consistent with the results of this study.

### Anxiety and depression were negatively correlated with prospective and retrospective memory and academic delay of gratification

The path analysis further confirmed that GAD-7 and PHQ-9 had a effect on ADOG. One possible reason for this might be that students with less anxiety and depression focused on learning, eliminating the interference of other external adverse factors, and were able to keep themselves in a balanced and stable psychological state. This was conducive for the improvement of the learning interest and efficiency. The students with severe anxiety and depression had decreased learning self-confidence, exhibited physical discomfort, inability to concentrate, weakened attention to learning and affected ADOG scores as well as worsened academic performance.

The path analysis of this study found that GAD-7 and PHQ-9 affected the ADOG scores through PRM. Previous studies have found that negative emotions such as anxiety and depression affected prospective memory performance (Harris and Cumming, 2003; Schnitzspahn et al., 2014). Negative emotions hindered the development of the prospective memory, while positive emotions promoted the improvement of prospective memory performance (Harris and Menzies, 1999). Some study found that patients with depression had impairment of the prospective and retrospective memory (McFarland and Vasterling, 2018). The reason why anxiety and depression affected the prospective memory might be that the adverse emotions affected the allocation of cognitive resources. Individuals needed to increase the allocation of resources to process emotion-related information, to reduce the psychological resources used for the current task, thus affecting the performance of prospective memory (Xian et al., 2020).

### Prospective and retrospective memory was positively correlated with academic delay of gratification

Prospective memory refers to the memory of plans. After receiving the memory task, the memory of plans is in the state of subliminal activation, which is extracted and put into action at a specific time. In this process, through cognitive monitoring, individuals perceive the passage of time, monitor the emergence of target clues, interrupt the interference task in time, and transfer their attention to the prospective memory task (Matos and Albuquerque, 2021). Prospective memory includes setting goals, clarifying the priority of goals, evaluating and monitoring the implementation of goals, and terminating inappropriate interference with goals in time (Bembenutty and Karabenick, 1998). Therefore, both PRM and prospective memory are related to self-control and self-regulation to achieve plans.

Prospective memory is an important cognitive activity closely related to people’s daily lives. In recent years, with the continuous promotion and innovation of research methods, prospective memory has become a hot topic in the field of cognitive research (Im et al., 2020).

Although no studies have found that prospective memory directly affects ADOG, some studies have confirmed that prospective memory may affect ADOG in other ways. For example, episodic future thinking has been confirmed as an effective way to counter impulsive behavior and decisions by adding weights to delayed benefits (Peters and Büchel, 2010; Benoit et al., 2011; Daniel et al., 2013). Damage to the brain area that is responsible for episodic future thinking was found to affect the use of episodic future thinking in making more future-oriented decisions (Palombo et al., 2015). Additionally, studies have found that future time insight is related to prospective memory and could predict the ability of ADOG, which supports the effect of prospective memory on ADOG (Yan, 2011).

Therefore, combined with the results of the chain mediation analysis, we speculate that the effect of gender on ADOG might have been caused by the anxiety and depression-induced impairment of prospective memory, which led to the reduction in ADOG.

The study results provide important information that can be applied to related theories and preventive interventions for improving ADOG in college students. First, this study raised awareness of the different mental health responses of male and female college students to the external environment. Since male’s academic performance is more affected by environmental changes than female’s, improving male’s mental health could be one way to reduce the learning gap between male and female. Second, this study also determined that the mental health level of college students is lower than that of ordinary adults. Therefore, it is suggested that schools should provide corresponding psychological counseling to help students to improve their mental health. This will help them to improve prospective memory and academic performance. Third, the results provide a theoretical basis for improving the ADOG level by increasing prospective memory. Cognitive control and prospective memory in complex dynamic environments require cognitive flexibility (Boag et al., 2019). Thus, improving cognitive flexibility can improve prospective memory and promote the ability to delay gratification (Koslov et al., 2019). Whereas negative emotions such as anxiety and depression can reduce cognitive flexibility (Park and Moghaddam, 2017; Hsieh and Lin, 2019; Yu et al., 2020), which leads to decreased prospective memory. Consequently, psychological treatment, such as Acceptance and Commitment Therapy, can reduce anxiety and depression by improving psychological flexibility (Twohig and Levin, 2017). This provides a good way to improve prospective memory through psychotherapy.

Limitations: First, as a cross-sectional correlation study, this study cannot determine the causal relationship, and subsequent experimental research is needed to further verify the hypothesis model. Second, many factors influence ADOG among college students, and there may be mechanisms involving other variables that affect the relationship between gender and ADOG. Third, the data was based on subjective self-rating scales, which may result in deviations. Thus, future research should include a combination of evaluations to increase objectivity. Overall, further research on the mechanisms of gender that affect students’ prospective memory, ADOG, and academic achievement has important reference value for constructing theoretical learning models and exploring ways to cultivate and improve ADOG.

## Data availability statement

The original contributions presented in the study are included in the article/supplementary material, further inquiries can be directed to the corresponding author.

## Ethics statement

The studies involving human participants were reviewed and approved by Institutional Review Board of Bengbu Medical College. The patients/participants provided their written informed consent to participate in this study.

## Author contributions

C-YJ and D-LJ conceived and designed the experiments. XS and L-PF carried out experiments. XS analyzed experimental data. C-YJ wrote the first draft of the manuscript. L-PF and D-LJ provided critical revision of the manuscript for important intellectual content. All authors have materially participated in the manuscript preparation.

## Funding

This project was supported by Innovative Training Program for Chinese College Students (Nos. 202210367035 and S202110367098), Shanghai Key Laboratory of Psychotic Disorders Open Grant (No. 13dz2260500), Bengbu Medical College key Laboratory of Addiction Medicine 29–3, and Teaching Research Project of Anhui Province (2020jyxm1183).

## Conflict of interest

The authors declare that the research was conducted in the absence of any commercial or financial relationships that could be construed as a potential conflict of interest.

## Publisher’s note

All claims expressed in this article are solely those of the authors and do not necessarily represent those of their affiliated organizations, or those of the publisher, the editors and the reviewers. Any product that may be evaluated in this article, or claim that may be made by its manufacturer, is not guaranteed or endorsed by the publisher.
